# Sex difference in the interaction of alcohol intake, hepatitis B virus, and hepatitis C virus on the risk of cirrhosis

**DOI:** 10.1371/journal.pone.0185710

**Published:** 2017-11-15

**Authors:** Tommaso Stroffolini, Evangelista Sagnelli, Angelo Andriulli, Guido Colloredo, Caterina Furlan, Giovanni Battista Gaeta, Filomena Morisco, Mario Pirisi, Floriano Rosina, Caterina Sagnelli, Antonina Smedile, Piero Luigi Almasio, On behalf of EPACRON study group

**Affiliations:** 1 Department of Tropical and Infectious Diseases, Policlinico Umberto I, Rome, Italy; 2 Department of Mental Health and Public Medicine, Campania University Luigi Vanvitelli, Naples, Italy; 3 Division of Gastroenterology, Casa Sollievo Sofferenza Hospital, IRCCS, San Giovanni Rotondo, Italy; 4 Department of Internal Medicine, San Pietro Hospital, Ponte San Pietro, Italy; 5 Infectious Diseases, Department of Mental and Physical Health and Preventive Medicine, Campania University Luigi Vanvitelli, Naples, Italy; 6 Department of Clinical Medicine and Surgery, Gastroenterology Unit, University of Naples “Federico II”, Naples, Italy; 7 Department of Translational Medicine, Università degli Studi del Piemonte Orientale "A. Avogadro", Novara, Italy; 8 Liver Unit, Hospital ''G. Garibaldi" Catania, Catania, Italy; 9 Liver Physiopathology Lab, Department of Medical Sciences, University of Turin, Turin, Italy; 10 Biomedical Department of Internal Medicine e Specialities (Di.Bi.M.I.S.), University of Palermo, Palermo, Italy; Kaohsiung Medical University Chung Ho Memorial Hospital, TAIWAN

## Abstract

**Background:**

The joint effect of the interaction of alcohol intake, hepatitis B virus (HBV) and hepatitis C virus (HCV) on the risk of cirrhosis is still unexplored because a large sample size is required for this investigation.

**Objective:**

Evaluation of interaction of HBV, HCV and alcohol abuse on the risk of cirrhosis.

**Design:**

We analysed 12,262 consecutive patients with chronic liver disease of various aetiologies referring to 95 Italian liver units in 2001 or 2014. To evaluate the interaction between alcohol abuse, HBV infection, and HCV infection, patients unexposed to either factors were used as reference category. Adjustment for BMI and age was done by multiple logistic regression analysis.

**Results:**

Females were older than males (p<0.01) and less frequently showed HBV and alcoholic aetiology (p<0.01). In both sexes, an overtime increasing age and an increasing proportion of subjects with liver cirrhosis was observed, reflecting a better survival (0.01).

An additive interaction is observed in females: the O.R. generated by the simultaneous presence of HBV, HCV, and alcohol (5.09; 95% C.I. 1.06–24.56) exceeds the sum (4.14) of the O.R. generated by a single exposure (O.R. = 0.72 for HBsAg positivity, OR = 1.34 for anti-HCV positivity, and O.R. = 2.08 for alcohol intake). No interaction is observed in male sex.

**Conclusions:**

The observed gender difference suggests that the simultaneous presence of HBV/HCV coinfection and risky alcohol intake enhances the mechanism of liver damage to a greater extent in females than in males.

## Introduction

Chronic hepatitis B (HBV) infection, chronic hepatitis C virus (HCV) infection, and risky alcohol intake are the main risk factors for chronic liver disease worldwide. Chronic hepatitis due to dual HBV/HCV infection more likely progresses to liver cirrhosis as compared to patients with single viral infection [1–4]. Besides, in subjects with a risky alcohol intake, coexistence of HCV infection [5–9] or HBV infection [9] strongly increases the risk of cirrhosis development. Studies assessing the effect of coexistence of HBV, HCV, and alcohol intake on the progression of chronic hepatitis to liver cirrhosis are lacking, most probably because the simultaneous presence of these three factors in the same patient is infrequent and thus large sample size is required to prove such potential effect.

Pooling the cases recruited in two national surveys [10,11] performed in Italy in 2001 and 2014, to assess clinical features of chronic liver diseases, we gathered a total sample of 12,262 subjects. This large sample size allows us the opportunity for estimating the interactions, if any, between alcohol intake, HBV infection, and HCV infection on the risk of cirrhosis by gender.

## Materials and methods

### Ethics statement

This study (Extensive protocol, participating centers and summary grid) was formally and definitively approved by the Ethics Committee of the Coordinating Center (Prof. Piero Luigi Almasio), Ethics Committee of the University Hospital (Azienda Ospedaliera Universitaria-Policlinico) "P. Giaccone" of Palermo, Italy, on December 9, 2015 (Verbal number 11/2015, point 18.)

All procedures applied in the study were in accordance with the international guidelines, with the standards of human experimentation of the local Ethics Committees and with the Helsinki Declaration of 1975, revised in1983.

At the time of the first observation, each patient signed an informed consent for the collection of personal data, as designated by the Ethics Committee of the coordinating centre. Patients who agreed to undergo liver biopsy signed an appropriate informed consent before biopsy was performed.

The collection of personal data was made in full compliance with the Italian law on personal data protection, and each patient gave his/her informed consent to participate.

### Patients

The two national surveys have been previously described [10,11]. The first one enrolled all persons consecutively referring to 79 liver units for a six months’ period in 2001 and the second one recruited all subjects referring to 16 hospitals liver units in 2014. Criteria of enrolment were age over 18 years and altered either hepatic biochemistry or presence of etiologic markers of liver damage or symptoms consistent with chronic liver disease. In both studies, the enrolling liver units were scattered all over the country. Many of the 16 hospitals participating in the second study had also taken part in the first one.

All patients were included only once at the time of first observation. For each patient, a pre-coded questionnaire containing demographic, epidemiological and clinical data was filled out. No patient refused to participate in the study.

The presence of serum HBsAg identified an HBV aetiology; the detection of anti-HCV an HCV aetiology. Autoimmune chronic hepatitis and primary biliary cirrhosis were diagnosed according to standardized international criteria [12,13]. The diagnosis of hereditary hemochromatosis was based on abnormal ferritin serum values and transferrin saturation serum values, genetic markers, or liver histology [14]. Wilson’s disease was a rare diagnosis based on accepted criteria [15]. The presence of a metabolic syndrome was established based on accepted criteria [16]. Abnormal serum alanine aminotransferase (ALT) values and a histological and/or ultrasound pattern of hepatic steatosis, in the absence of other known causes of chronic liver disease, were considered related to non-alcoholic fatty liver disease [13]. Alcohol intake ≥40 g/day for males and ≥30 g/day for females for at least 5 years was considered an etiologic factor of liver disease [17]. The diagnosis of cryptogenic chronic liver disease was based on the absence of any viral, autoimmune or metabolic aetiology.

Chronic hepatitis was diagnosed based on liver histology, when available, or on the persistence (>6 months) of abnormal ALT in the absence of clinical, biochemical, and ultrasound markers of liver cirrhosis [18]. Liver cirrhosis was diagnosed by liver biopsy (LB) or on the presence of characteristic clinical, biochemical, and ultrasound signs [18]. The diagnosis of hepatocellular carcinoma (HCC) was based on histological and/or imaging findings and alfa-1-fetoprotein serum levels, according to accepted criteria [19]. Serum HBsAg and antibody to HCV, HDV and HIV were sought using commercial immunoenzymatic assays. The etiologic markers of autoimmune hepatitis, PBC, iron and copper overload and liver function were assessed by routine tests.

### Statistical analysis

Crude O.R. were adjusted for the confounding effect of age and B.M.I. by multiple logistic regression analysis. Interaction between risk factors for a given outcome may be additive or multiplicative. Additive interaction, means that the observed joint effect of two or more factors on the disease incidence (in this case cirrhosis), exceeds the sum of the effect of exposure to each single factor minus 1. Multiplicative interaction means that the observed joint effect of factors on the disease incidence exceeds the product of the effect of exposure to each single factor.

To evaluate the interaction between alcohol abuse, HBV infection, and HCV infection, patients enrolled in the 2001 and 2014 surveys and unexposed to these three factors were taken as the reference category for all calculations. Thus, Reference category was represented by subjects with other causes of chronic liver disease (NASH, autoimmunity, genetics, and so on). We dichotomized alcohol intake as <3 units/day vs. ≥ 3 units/day in men, and <2 units/day vs. ≥2 units/day in women.

## Results

Overall, 12,262 subjects were enrolled. Their baseline characteristics by sex are reported in Table 1. The sex ratio (M/F) was 1.4. Females were more likely older (Mean age 58.7 y. vs. 52.9 y; p<0.01). Significant inter gender differences were observed according to the three risk factors considered. The baseline characteristics of subjects by sex and year of recruitment (Table 2) shows in both sexes an increasing age and an increasing proportion of subjects with liver cirrhosis, reflecting a better survival.

**Table 1:** Baseline characteristics of 12,262 enrolled subjects by sex.

Variables	Male (N = 7,138)	Female (N = 5,124)	p-value
**Age, years M±SD**	52.9±15.1	58.7±13.7	<0.01
**BMI, kg/m**^**2**^ **M±SD**	25.4±3.5	25.1±4.2	N.S.
**Risk factors, N (%):**			<0.01
**-HBsAg positive alone or with other factor**	1,265 (17.8)	559 (10.9)	
**-Anti-HCV positive alone or with other factor**	4,647 (65.1)	3,812 (74.4)	
**-Alcohol intake alone or with other factor** *	1,962 (29.0)	658 (12.9)	
**Diagnosis, N (%):**			N.S.
**-Chronic hepatitis**	5,302 (74.3)	3,808 (74.3)	
**-Liver cirrhosis without HCC**	1,528 (21.4)	1,139 (22.2)	
**-Liver cirrhosis with HCC**	308 (4.3)	177 (3.5)	

*: ≥ 3 drinks a day for male and ≥ 2 drinks a day for females.

BMI: Body Max Index

HCC: hepatocellular carcinoma

HCC: hepatocellular carcinoma

**Table 2:** Baseline characteristics of 12,262 enrolled subjects by sex and years of study.

Variables	2001 Study (N = 9,752)			2014 Study (n = 2,510)		
	Male (N = 5,649)	Female (N = 4,103)	p-value	Male (N = 1,489)	Female (N = 1,021)	p-value
**Age, years M±SD**	51.7**±**15.2	58.1**±**13.	<0.01	57.3**±**14.1	61.3**±**13.1	<0.01
**BMI, kg/m**^**2**^ **M±SD**	25.5**±**3.4	25.0**±**4.1	N.S.	26.3**±**3.9	25.7**±**4.6	<0.05
**Risk factors, N (%):**						
**- HBsAg positive alone or with other factor**	934 (16.5)	377 (3.2)	<0.01	332 (22.3)	182 (17.8)	<0.01
**-Anti-HCV positive alone or with other factor**	3,747 (66.3)	3,167 (77.2)		900 (60.4)	645 (63.2)	
**-Alcohol intake alone or with other factor** *	1,852 (32.8)	490 (11.5)		211 (14.2)	168 (16.5)	
**Diagnosis, N (%)**						
**-Chronic hepatitis**	4,327 (76.6)	3,138 (76.5)	N.S.	975 (65.5)	670 (65.7)	N.S.
**-Liver cirrhosis without HCC**	1,102 (19.5)	849 (20.7)		426 (28.6)	290 (28.4)	
**-Liver cirrhosis with HCC**	220 (3.9)	116 (2.8)		88 (5.9)	61 (6.0)	

*: ≥ 3 drinks a day for male and ≥ 2 drinks a day for females.

BMI: Body Max Index

HCC: hepatocellular carcinoma

We tested the hypothesis of an additive interaction due to the simultaneous presence of HBV, HCV, and risky alcohol intake on the risk of progression to liver cirrhosis by sex. No additive interaction was observed in males since O.R. for cirrhosis (adjusted for B.M.I. and age) due to the joint exposure to HBsAg positivity, HCV positivity and ≥ 3 alcohol unit/day was 3.56 (95% C.I. = 1.92–6.62), a value lower than the sum (5.32) of each single exposure (1.39 for HBsAg positivity, 1,17 for HCV positivity and 2.85 for risky alcohol intake) minus 1 (Table 3).

**Table 3:** Interaction between alcohol intake and co-factors in the progression from chronic hepatitis to cirrhosis in male patients. Crude and Adjusted* Odds Ratio derived from multiple logistic regression analysis.

Variable	Absence of Cirrhosis N (%)	Cirrhosis/HCC N (%)	Crude OR (95% CI)	Adjusted OR* (95% CI)
**HBsAg/HCV/alcohol:**				
**HBsAg-/anti-HCV-/Alcohol intake < 3 units**	490 (82.5%)	104 (17.5%)	1	1
**HBsAg-/anti-HCV-/Alcohol intake ≥ 3 units**	487 (60.0%)	324 (40.0%)	3.1 (2.4–4.0)	2.9 (2.2–3.8)
**HBsAg +/anti-HCV-/Alcohol intake <3 units**	731 (77.8%)	208 (22.2%)	1.3 (1.0–1.7)	1.4 (1.0–1.9)
**HBsAg-/anti-HCV+/Alcohol intake < 3 units**	2,655 (77.5%)	771 (22.5%)	1.4 (1.1–1.7)	1.2 (0.9–1.5)
HBsAg+/ant-HCV+/Alcohol intake ≥ 3 units**	40 (64.5%)	22 (35.5%)	2.6 (1.5–4.5)	3.6 (1.9–6.6)

*Adjusted for the confounding effect of age (<53 vs. ≥ 53 years) and BMI (<30 vs. ≥30).

**No additive interaction is observed

In contrast, an additive interaction was observed in female sex. In fact, O.R. for cirrhosis due to the joint exposure to the three risk factors was 5.09 (95% C.I. = 1.06–24.56), a value exceeding the sum (4.14) of the O.R. for cirrhosis of each single exposure (0.72, for HBsAg positivity; 1,34, for HCV positivity and 2.08 for risky alcohol intake) minus 1 (Table 4).

**Table 4:** Interaction between alcohol intake and co-factors in the progression from chronic hepatitis to cirrhosis in female patients. Crude and Adjusted Odds* Ratio derived from multiple logistic regression analysis.

Variable	Absence of Cirrhosis N (%)	Cirrhosis/HCC N (%)	Crude OR (95% CI)	Adjusted OR* (95% CI)
**HBsAg/HCV/alcohol**				
**HBsAg-/anti-HCV-/Alcohol intake < 2 units**	350 (75.4%)	114 (24.6%)	1	1
**HBsAg-/anti-HCV-/Alcohol intake ≥ 2 units**	217 (61.6%)	135 (38.4%)	1.9 (1.4–2.6)	2.1 (1.5–2.9)
**HBsAg+/anti-HCV-/Alcohol intake < 2 units**	387 (82.2%)	84 (17.8%)	0.7 (0.5–0.9)	0.7 (0.5–1.0)
**HBsAg-/anti-HCV+/Alcohol intake < 2 units**	2,620 (75.3%)	858 (24.7%)	1.0 (0.8–1.3)	1.3 (0.7–1.1)
HBsAg+/anti-HCV+/Alcohol intake ≥ 2 units**	4 (40.0%)	6 (60.0%)	4.6 (1.3–16.6)	5.1 (1.1–24.6)

*Adjusted for the confounding effect of age (<59 vs. ≥ 59 years) and BMI (<30 vs. ≥30).

**An additive interaction is observed

No additive interaction was observed when the two genders are combined for analysis: O.R. 3.3 for the joint presence of the three risk factors, but O.R. 4.5 for the sum of each separate risk factor (data no shown).

## Discussion

Several liver units have participated in both 2001 and 2014 surveys using comparable access procedures, similar clinical approach and comparable methods. As consequence, the pool of the two series of cases (2001 and 2014) may not be matter of concern.

To evaluate the interaction between alcohol intake and HBV/HCV infection on the risk of cirrhosis we have used an internal control, which is the best control group. Cases (i.e. cirrhotic patients) and controls (i.e. chronic hepatitis cases) were subjects extracted from the same population (i.e. subjects with chronic liver disease enrolled in 2001 and 2014) and thus exposed to the same potential selective factors. In a case-control study, the comparability between cases and controls is a crucial factor, which ensures the avoidance of potential selective factors regarding the enrolment of cases and controls. Thus, the use of an internal control group may have avoided spurious associations.

We acknowledge that the use as reference category of subjects with chronic liver disease not related to HBV, HCV or risky alcohol intake might have underestimated the true effect on cirrhosis development of both single and simultaneous exposures evaluated in this study. For example, the adjusted OR of cirrhosis in HBsAg positive females (anti-HCV negative/Alcohol intake<2 Units) is only 0.7, suggesting that this category of subjects is not at higher risk of cirrhosis than those with other causes of chronic liver diseases (NASH, autoimmunity, genetics and so on) enclosed in the reference category. The aim of this study, however, was not to estimate the strength of association linking alcohol, HBV, and HCV with cirrhosis (data well known), but to assess the interaction, if any, due to the contemporaneous presence of these three exposures on the risk of cirrhosis. This aim is not affected by the type of reference category chosen; but the magnitude of the risk results likely underestimated.

The increase in the rate of HBV-related cases observed in 2014 may be due to a referral bias, since after 2001 highly effective treatments (adefovir plus lamivudine, entecavir and tenofovir) for long-term suppression of HBV replication were issued free of charge in hospital centres. The decrease of the impact of alcohol-related etiology, is attributable to the dramatic increase in the rate of male abstainers, in line with the consistent reduction in alcohol abuse in the last decade in Italy consequent to both to the efforts of the Italian Health Authorities to prevent alcohol abuse and to the economic crisis of our Country reducing the likelihood of purchasing alcoholic beverages in in the last decade.

It is well known that NASH is an important risk factor for liver cirrhosis. Adjustment for this potential confounder has not been made, because this category of subjects was enclosed in the reference category.

The present findings evidence that the combined presence of risky alcohol intake, HBV, and HCV generates an additive effect on the risk of cirrhosis in females. Some lines of evidence provide the biological plausibility for this finding. Firstly, both hepatitis viruses and alcohol may stimulate hepatic-oxidative stress, which leads to the activation of liver fibrogenic cells, and consequent acceleration of fibrogenesis [20]. Secondly, immune response is affected by alcohol [21] that may also promote apoptosis in HCV infected hepatocytes [22]. There are conflicting reports on the interference of HBV infection with the apoptosis, most studies showing inhibition and a minority induction, the HBV-mediated alteration of apoptosis being prevalently achieved via interference with cellular signaling pathways and regulation of epigenetics HBV X protein (HBX) [23].

On the contrary, no additive effect was observed in males, as expected based on data showing a progression to liver cirrhosis of both HBV [24,25] and HCV [26,27] chronic hepatitis more likely in males than in females. This discrepancy may reflect a role of alcohol intake in enhancing the pathogenic mechanisms of liver damage, more likely in females than in males.

In conclusion, this study shows a sex difference in liver disease progression in subjects with simultaneous presence of HBV/HCV dual infection and history of alcohol abuse, suggesting that females are at higher risk of liver cirrhosis than males and deserve a more careful surveillance.

## Supporting information

S1 Dataset Data underlying this study. (XLS)
